# Ocular expressions of children behcet disease

**DOI:** 10.1186/1546-0096-12-S1-P235

**Published:** 2014-09-17

**Authors:** Bouchra Chkirate, Sabah Jaouhari, Abelali Bentahila

**Affiliations:** 1 Pediatric Hospital, University of Medicine, Rabat, Morocco

## Introduction

Behcet disease is a systemic vascularitis, the diagnosis is difficult in child. The most frequent ocular signs in child is panuveitis with retinian vascularitis.

## Objectives

The aim of this study is to clarify the frequency and clinical caracteristics of ocular attack in child behcet disease in our context.

## Methods

It’s a retrospective study concerning 13 cases of child behcet disease with ocular signs in service of pediatrie 4 and peadtric rheumatology consultation at Rabat children hospital during 5 years ( April 2007- January 2013 ).

We have studied the onset of symptoms, ocular anatomo clinical form, complications and therapeutical implications.

## Results

Our patients were 4 to 15 years old ( average 10,78 years ). Ocular signs were found at 13 cases among 19 followed for behcet disease ( 68,4% ). The attack was bilateral in 11 cases ( 84% ). In 2 cases, ocular attack was inaugural. The uveitis was total in 3 patients, anterior in 3 others cases, intermediar in 2 cases, severe in one case. Papillar oedema was noted in 2 cases, retinian vascularitis in 3 cases. 4 patients had ocular complications : 2 optic atrophie, 1 cataract and 1 macular oedema.

## Conclusion

Ocular signs in Behcet disease was found in 10 to 52,5% in pediatric series. They are often bilateral. Panuveitis and anterior uveitis are the most frequent signs followed in our serie by intermediar uveitis. This finding concord with a tunisian serie.

Visual prognosis is threaten by ocular complications witch are precocious in child : cataract, macula oedema, optic atrophie indeed blindness.

## Disclosure of interest

None declared.

